# Chronic primary adrenal insufficiency after unilateral adrenonephrectomy: A case report

**DOI:** 10.1097/MD.0000000000009091

**Published:** 2017-12-22

**Authors:** Satoshi Yoshiji, Kimitaka Shibue, Toshihito Fujii, Takeshi Usui, Keisho Hirota, Daisuke Taura, Mayumi Inoue, Masakatsu Sone, Akihiro Yasoda, Nobuya Inagaki

**Affiliations:** a Department of Diabetes, Endocrinology and Nutrition, Kyoto University Graduate School of Medicine, Kyoto; b Department of Medical Genetics, Shizuoka General Hospital, Shizuoka, Japan.

**Keywords:** adrenal stress, adrenalectomy, adrenonephrectomy, primary adrenal insufficiency, renal cell carcinoma

## Abstract

**Rationale:**

Unilateral adrenalectomy as part of surgical resection of renal cell carcinoma (RCC) is not thought to increase the risk of chronic adrenal insufficiency, as the contralateral adrenal gland is assumed to be capable of compensating for the lost function of the resected gland. However, recent studies have indicated that adrenalectomy might cause irreversible impairment of the adrenocortical reserve. We describe a case of chronic primary adrenal insufficiency in a 68-year-old man who previously underwent unilateral adrenonephrectomy, which was complicated by severe postoperative adrenal stress that involved cardiopulmonary disturbance and systemic infection.

**Patient concerns:**

A 68-year-old Japanese man presented with weight loss of 6 kg over a 4-month period, and renal biopsy confirmed a diagnosis of RCC. He underwent adrenonephrectomy for the RCC, but developed postoperative septic shock because of a retroperitoneal cystic infection and ventricular fibrillation that was induced by vasospastic angina. The patient was successfully treated using antibiotics and percutaneous coronary intervention, and was subsequently discharged with no apparent complications except decreased appetite and general fatigue. However, his appetite and fatigue did not improve over time and he was readmitted for an examination.

**Diagnoses:**

The workup revealed a markedly elevated adrenocorticotropic hormone (ACTH) level (151.4 pg/mL, normal: 7–50 pg/mL) and a mildly decreased morning serum cortisol level (6.4 mg/mL, normal: 7–28 mg/mL). In addition to the patient's clinical symptoms and laboratory results, the results from ACTH and corticotropin-releasing hormone stimulation tests were used to make a diagnosis of primary adrenal insufficiency.

**Interventions:**

Treatment was initiated using oral prednisolone (20 mg), which rapidly resolved his symptoms. At the 1-year follow-up, the patient had a markedly decreased serum cortisol level (2.0 mg/mL) with an ACTH level that was within the normal range (44.1 pg/mL) before his morning dose of prednisolone, which confirmed the diagnosis of chronic primary adrenal insufficiency.

**Lessons:**

Clinicians must be aware of chronic adrenal insufficiency as a possible complication of unilateral adrenalectomy, especially when patients who underwent unilateral adrenalectomy experience severe adrenal stress.

## 1 Introduction

Unilateral adrenalectomy is not thought to increase the risk of adrenal insufficiency,^[1]^ and chronic postoperative adrenal insufficiency after unilateral adrenalectomy has rarely been reported. This is thought to be because the contralateral adrenal gland can compensate for the lost function of the resected gland.^[2–4]^ Therefore, unilateral adrenalectomy is not expected to cause chronic adrenal insufficiency in normal settings. However, even patients with 2 healthy adrenal glands can develop adrenal insufficiency during periods of severe adrenal stress.^[5,6]^ Thus, it is unsurprising that adrenal insufficiency could occur after unilateral adrenalectomy and severe adrenal stress, although it is problematic that postoperative adrenal insufficiency may be overlooked in cases of unilateral adrenal insufficiency. We report a case of chronic primary adrenal insufficiency in a 68-year-old man who underwent unilateral adrenonephrectomy, which was followed by severe postoperative adrenal stress consisting of cardiopulmonary disturbance and systemic infection.

## 2 Case presentation

A 68-year-old Japanese man presented with weight loss of 6 kg over a 4-month period. His other medical problems included hypertension and hyperuricemia. The patient's family history was unremarkable. Physical examination revealed conjunctival pallor. Computed tomography (CT) revealed a 7-cm left renal mass, and renal biopsy confirmed a diagnosis of renal cell carcinoma (RCC), although no apparent adrenal metastasis was detected during the CT. Nevertheless, the patient underwent unilateral nephrectomy with ipsilateral adrenalectomy based on the current guidelines for RCC. The guidelines recommend ipsilateral adrenalectomy with radical nephrectomy for tumors that are >6 cm, based on their high risk of adrenal metastasis, even in the absence of radiographic evidence of metastasis.^[7]^

The surgery was uneventful, although the patient lost consciousness when he stood up for the first time on postoperative day (POD) 1. The patient's blood pressure could not be measured, and electrocardiography revealed ventricular fibrillation. The patient received 4 injections of epinephrine and 2 defibrillator shocks, which led to the return of spontaneous circulation at approximately 20 minutes after the ventricular fibrillation was detected. Pulmonary embolism was initially suspected, although CT failed to detect any emboli. The workup included coronary angiography, which revealed 90% luminal narrowing of the #6 branch of the lateral ascending artery, and the narrowing was attributed to vasospastic angina. The patient underwent percutaneous coronary intervention, which successfully stabilized his cardiopulmonary condition. Laboratory testing revealed marked increases in potassium levels and eosinophil counts from POD 1 to POD 2 (Table 1), when the patient experienced the cardiopulmonary disturbance.

**Table 1:**
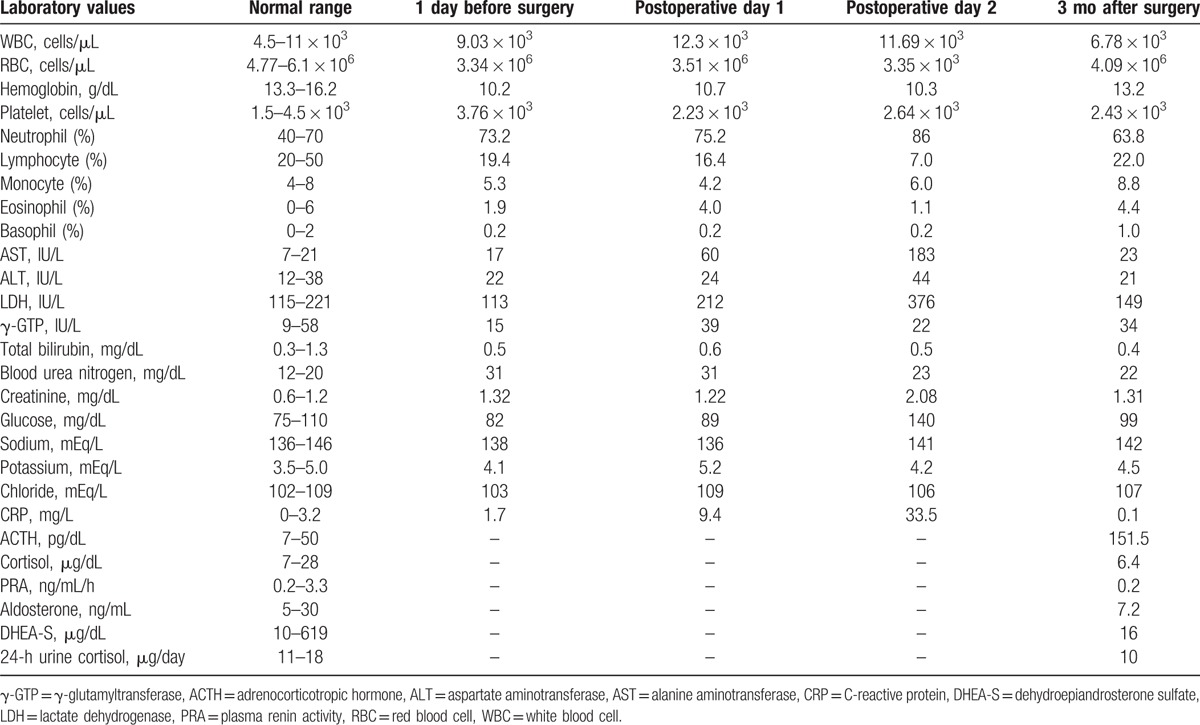
The patient's laboratory results during perioperative period and follow-up.

Two weeks after the percutaneous coronary intervention, the patient developed a retroperitoneal cystic infection, which induced fever and hypotension, and *Pseudomonas aeruginosa* was identified as the causative agent. He was successfully treated using meropenem and intravenous fluid, recovered without any further adverse events, and was discharged at 2 months after the adrenonephrectomy. At the time of discharge, he still had decreased appetite and general fatigue. However, the patient's fatigue and appetite did not improve with rest at home, and he was readmitted to our center at 1 month after his discharge (3 months after the surgery). The workup revealed a markedly elevated morning serum level of adrenocorticotropic hormone (ACTH; 151.4 pg/mL, normal: 7–50 pg/mL) and a mildly decreased morning serum cortisol level (6.4 μg/mL, normal: 7–28 μg/mL) (Table 2).

**Table 2:**
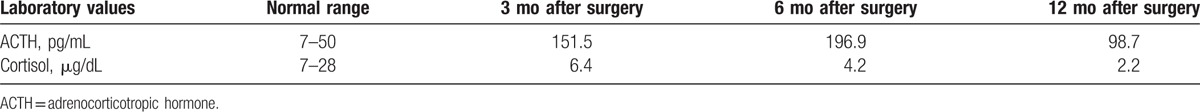
Serial change in adrenocorticotropic hormone and cortisol.

**Figure 1:**
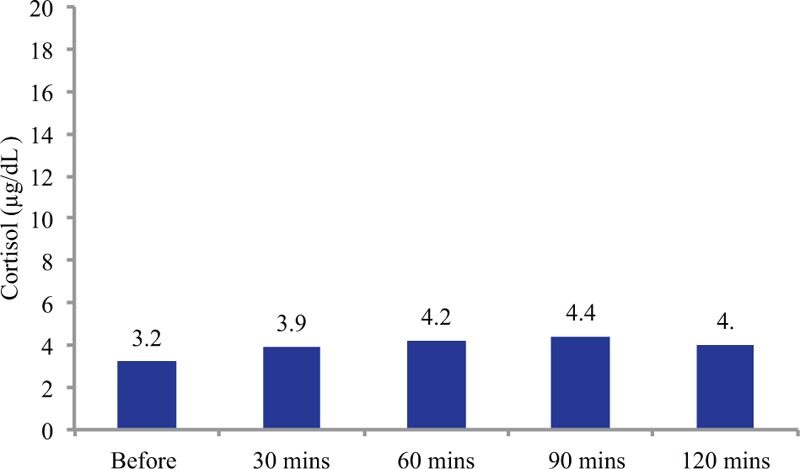
The adrenocorticotropic hormone stimulation test results. Intravenous adrenocorticotropic hormone (250 μg) did not increase the serum cortisol levels above 4.4 μg/dL, which is far below the standard range (≥18–20 μg/dL).

**Figure 2:**
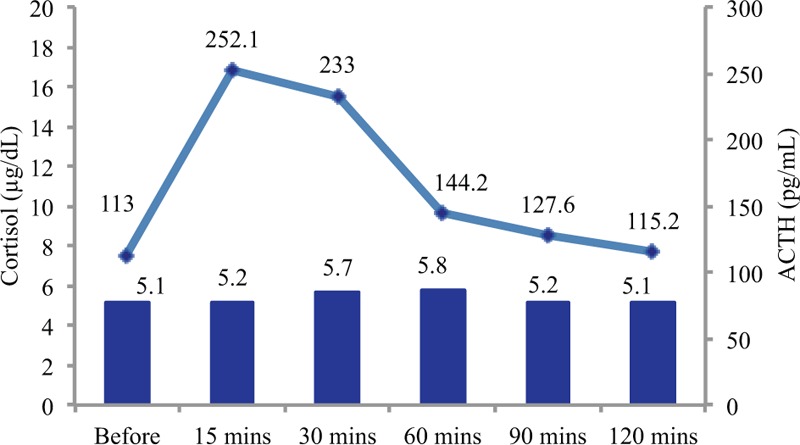
The corticotropin-releasing hormone stimulation test results. The bars reflect the cortisol levels and the line reflects the adrenocorticotropic hormone (ACTH) levels. After the corticotropin-releasing hormone stimulation test, the ACTH level rose significantly from 113 to 252.1 pg/dL. However, the serum cortisol levels barely increased after the stimulation, from 5.1 to 5.8 μg/dL (normal: ≥18–20 μg/dL).

The patient underwent ACTH and corticotropin-releasing hormone stimulation tests, which both revealed markedly decreased responses (Figs. 1 and 2, respectively). His plasma renin activity, aldosterone level, and dehydroepiandrosterone sulfate (DHEA-S) levels were all within the normal limits (Table 1). Postoperative CT of the remaining adrenal gland, magnetic resonance imaging of the head, and histopathological analysis of the resected gland revealed no abnormalities. On the basis of the patient's laboratory data and his clinical symptoms, primary adrenal insufficiency was suspected, and treatment was started using oral prednisolone (20 mg). The patient's appetite and fatigue significantly improved shortly after the initiation of prednisolone treatment.

To exclude 21-hydroxylase deficiency as an underlying disease, genetic analysis of the *CYP21A2* gene was performed, although no mutations were observed. Testing at 6 months after the surgery, and before the morning prednisolone dose, revealed a lower morning serum cortisol level (2.2 μg/mL) and an elevated morning serum ACTH level (98.7 pg/mL). At the 1-year follow-up, the patient's morning cortisol level was 2.0 μg/mL and his ACTH level was 44.1 pg/mL before the morning dose of prednisolone, which was consistent with the diagnosis of chronic primary adrenal insufficiency.

The patient gave written informed consent. The ethical approval was not required because of the retrospective nature of the case report.

## 3 Discussion

Unilateral adrenalectomy is not thought to increase the risk of chronic adrenal insufficiency in patients with normal adrenocortical function.^[1]^ This is because the contralateral gland has sufficient capacity to compensate for the lost function of the resected gland in normal settings. Although hypothalamic-pituitary-adrenal suppression can be caused by cortisol-producing adrenocortical tumors, even in patients with preoperative hypercortisolism (e.g., clinical and subclinical Cushing syndrome), unilateral adrenalectomy rarely causes long-term irreversible adrenal insufficiency.^[3]^ However, Yokoyama and Tanaka^[2]^ have reported that unilateral adrenalectomy might cause irreversible impairment of adrenocortical function, and that patients had markedly elevated ACTH levels and normal serum cortisol levels after ipsilateral adrenalectomy and radical nephrectomy for RCC. Honda et al^[4]^ also reported that basal ACTH levels were elevated after unilateral adrenalectomy, although basal cortisol levels were sustained, and that the adrenal reserve function determined using the ACTH stimulation test was significantly attenuated. These findings indicate that patients who undergo unilateral adrenalectomy might have a decreased adrenocortical reserve, which can be compensated for by increased postoperative ACTH stimulation from the pituitary gland. Furthermore, sepsis, cardiopulmonary disturbance, trauma, and various other factors can cause postoperative adrenal insufficiency.^[8,9]^ Thus, during periods of severe postoperative adrenal stress, patients with 1 adrenal gland may be more likely to develop adrenal insufficiency because of a decreased adrenocortical reserve, compared with patients with 2 adrenal glands.

In the present case, the patient underwent unilateral adrenalectomy and subsequently developed cardiopulmonary disturbance and sepsis. It is possible that the unilateral adrenalectomy impaired his adrenocortical reserve, and that the postoperative cardiopulmonary disturbance and sepsis caused overwhelming damage to the remaining adrenal gland and led to the adrenal insufficiency. Laboratory testing revealed marked increases in potassium levels and eosinophil counts from POD 1 to POD 2 (Table 1), when the patient experienced the cardiopulmonary disturbance. This may indicate the development of adrenal insufficiency during that period, as the patient had decreased adrenocortical reserve and experienced overwhelming adrenal stress. Furthermore, a recent study from the Cleveland clinic indicated that postoperative adrenal insufficiency after unilateral adrenalectomy was unexpectedly common, and Mitchell et al^[10]^ found that 22% of patients without preoperative cortisol hypersecretion developed postoperative adrenal insufficiency after unilateral adrenalectomy. These findings suggest that clinicians should be aware that postoperative adrenal insufficiency after unilateral adrenalectomy is not as rare as previously thought, and that unilateral adrenalectomy must be performed with greater caution.

This case has several limitations. It remains unclear why our patient only had decreased serum cortisol levels while his serum aldosterone and DHEA-S levels were maintained within the normal ranges. It is possible that the severe adrenal stress (transient cardiopulmonary arrest and systemic infection) induced significant hypoxia and irreversible damage in the zona fasciculata, with relatively small effects on the zona glomerulosa and zona reticulata, which produce aldosterone and DHEA-S, respectively.^[10]^ The primary cause of the adrenal insufficiency might have been Waterhouse–Friderichsen syndrome, although the patient's relatively old age, causative bacteria for the sepsis (*P. aeruginosa*), and negative postoperative and follow-up CT findings make this diagnosis less likely.^[11,12]^ It is also possible that the patient had an underlying adrenal condition that predisposed him to adrenal insufficiency. For example, 21-hydroxylase deficiency is involved in >90% of congenital adrenal hyperplasia cases, which are associated with an inherited deficiency of 21-hydroxylase.^[13]^ However, we did not detect any mutations in the *CYP21A2* gene, which makes it unlikely that the patient had undiagnosed 21-hydroxylase deficiency. Given that most cases of congenital adrenal dysfunction with impaired cortisol secretion are attributable to 21-hydroxylase deficiency, it is unlikely that the patient had an undiagnosed congenital adrenal disorder.

## 4 Conclusion

Clinicians must be aware that chronic adrenal insufficiency is a possible complication of unilateral adrenalectomy, especially when patients who underwent unilateral adrenalectomy experience severe adrenal stress.
